# Effects of knee muscle fatigue on gait and proprioception in multiple sclerosis

**DOI:** 10.1590/1806-9282.20260933

**Published:** 2026-10-30

**Authors:** Muhammed Şeref Yıldırım, Cagla Ozkul, Gamze Cobanoglu, Kader Eldemir, Sefa Eldemir, Fettah Saygili, Nevin Atalay Guzel, Ceyla Irkec, Arzu Guclu-Gunduz

**Affiliations:** 1 Trakya University, Faculty of Health Sciences, Department of Physiotherapy and Rehabilitation – Edirne, Türkiye.; 2 Gazi University, Faculty of Health Sciences, Department of Physiotherapy and Rehabilitation – Ankara, Türkiye.; 3 Sivas Cumhuriyet University, Faculty of Health Sciences, Department of Physiotherapy and Rehabilitation – Sivas, Türkiye.; 4 Aydın Adnan Menderes University, Faculty of Health Sciences, Department of Physiotherapy and Rehabilitation – Aydın, Türkiye.; 5 Lokman Hekim University, Department of Neurology – Ankara, Türkiye.

**Keywords:** Fatigue, Gait, Multiple sclerosis, Proprioception, Rehabilitation, Strength

## Abstract

**OBJECTIVE:**

People with multiple sclerosis often experience muscle weakness and fatigue, which impair proprioception and gait. The effects of knee muscle fatigue on these outcomes remain unclear. This study investigated the effects of a knee muscle fatigue protocol on knee position sense and gait in people with multiple sclerosis.

**METHODS:**

A total of 36 mildly affected people with multiple sclerosis and nine healthy controls participated in this case-control study with repeated pre- and post-fatigue assessments. Gait was assessed using a G-Walk sensor, and knee position sense with an active repositioning test on an isokinetic dynamometer. Muscle strength and fatigue indexes were determined isokinetically for the knee flexors and extensors.

**RESULTS:**

During the fatigue protocol, people with multiple sclerosis produced lower total workloads than healthy control, although fatigue indexes were similar. After fatigue, both groups showed increased cadence and reduced gait cycle duration, while gait speed increased only in people with multiple sclerosis. Knee position sense deteriorated bilaterally in people with multiple sclerosis and on the non-dominant side in healthy control.

**CONCLUSION:**

Localized knee muscle fatigue altered knee position sense and gait in both groups, with no significant group×time interaction, though the small control group warrants caution. Similar fatigue indices despite lower total work warrant cautious interpretation of the Cybex fatigue index, given its dependence on baseline torque. As proprioceptive acuity declined transiently after localized knee muscle fatigue, clinicians may consider performing balance- or coordination-demanding tasks beforehand in people with multiple sclerosis.

## INTRODUCTION

Multiple sclerosis (MS) is a chronic demyelinating disorder of the central nervous system that frequently leads to progressive disability, with fatigue, muscle weakness, imbalance, and gait disorders significantly impacting daily life in people with multiple sclerosis (PwMS)^1^.

PwMS commonly exhibit reduced gait speed and stride length that worsen with increasing disability^2^. These gait abnormalities often coexist with knee flexor weakness and impaired balance control, contributing to walking-related functional decline in mildly affected PwMS^3^.

PwMS also commonly experience sensory impairments that influence gait, as joint position sense is essential for a smooth and coordinated gait pattern^4^. Due to its direct role in movement, knee position sense is a primary focus of research in PwMS^2,4-7^, though findings in mildly disabled patients remain inconsistent—some report no significant deficits^5,6^, while others demonstrate clear impairments^4,7^, with several linking position sense to gait parameters^2,4-6^.

Fatigue affects almost 80% of PwMS and often exacerbates gait and sensory impairments^8^. Lower extremity strength and position sense are linked to fatigue severity in PwMS^4^, fatigue induces meaningful spatiotemporal gait alterations^8^, and performance fatigability may occur even when self-reported fatigue is low^9^. Fatigue-related slowing has also been demonstrated during sustained tasks such as the 6-min walk test^10^.

This evidence, however, comes either from cross-sectional associations with perceived fatigue^4,9^ or from walking-based fatiguing tasks^8,10^. Motor fatigability is task-specific, and performance decline depends on the characteristics of the fatiguing task^11^. Among the available approaches, single-joint protocols stand out because the fatiguing stimulus can be standardized and quantified^11^, and among these, concentric lower-limb protocols reflect walking performance more closely than sustained isometric ones^12^. The knee flexors and extensors are of particular interest because proprioceptive input from their muscle spindles contributes to knee position sense^13^ and gait control. Together, these considerations support the use of a localized concentric knee flexor–extensor fatigue model to investigate acute changes in knee position sense and gait.

Despite this rationale, the specific effects of knee muscle fatigue on knee position sense and gait in PwMS remain understudied. This study investigated the effects of a standardized knee muscle fatigue protocol on (1) spatiotemporal gait characteristics and (2) knee position sense in PwMS versus healthy controls (HCs).

## METHODS

### Participants

This study was conducted at the Department of Physiotherapy and Rehabilitation, Gazi University, and included 36 PwMS and 9 HCs. Inclusion criteria were a diagnosis of MS per the revised McDonald criteria^14^, age 18–65 years, Expanded Disability Status Scale (EDSS) score <6^15^, and relapse-free status for ≥3 months. Exclusion criteria were a history of neurological disease other than MS and orthopedic, vision, or hearing impairments. Written informed consent was obtained from all participants. The study was approved by the Gazi University Clinical Research Ethics Committee, conducted per the Declaration of Helsinki, and registered at ClinicalTrials.gov (NCT04548310) before recruitment.

### Study design

This case-control study calculated the sample size (n=34 for PwMS) via power analysis (G*power version 3.1.9) with power=0.8 and alpha=0.05 to detect moderate within-group effects (dz=0.5)^16^. The calculation was based on the primary within-group analyses in PwMS; no separate calculation was performed for the HC group, which served as a reference group to contextualize baseline values and the acute response to the fatiguing protocol. Between-group comparisons were therefore considered exploratory. Demographic and disease-related data were recorded, and gait, knee position sense, and muscle strength were assessed at baseline. After the fatigue protocol, gait and knee joint position sense were reassessed.

### Measurements

Gait parameters were evaluated using the G-Walk sensor system (BTS, Italy), attached at L5 with an elastic belt. Participants walked along a 10-m track at their self-selected comfortable walking speed. Following two familiarization trials, one successful 10-m walking trial was recorded and used for analysis. Acceleration data were recorded at 100 Hz, analyzed with G-Studio, and spatiotemporal gait parameters were extracted^17^.

Knee joint position sense was assessed using an isokinetic dynamometer (Cybex Norm^®^, Humac, USA). The isokinetic dynamometer has demonstrated excellent reliability for angular position measurements (ICC≥0.99)^18^. A comparable 30° active knee repositioning protocol using the Cybex system has also been evaluated previously^19^, and the 30° target angle was selected as a functionally relevant angle within the 20–40° range associated with proprioceptive feedback during normal walking^20^. Participants were seated with the tested knee aligned to the dynamometer axis and blindfolded. The knee was passively moved from 90° flexion to a 30° target angle, held for 5 s, and then reproduced by the participant. Five trials were performed per knee, and mean absolute angular error was calculated for analysis^21^.

Knee muscle strength was assessed using the same isokinetic system. The dynamometer was routinely calibrated biweekly, and a pre-measurement calibration was performed before testing. Participants were seated with the hips flexed to 90° and the trunk inclined to 85°, with the dynamometer axis aligned to the lateral femoral condyle. Stabilization straps were applied across both shoulders, the waist, and the tested thigh, and the ankle strap was positioned 3 cm proximal to the lateral malleolus. Automatic gravity correction was performed using the system software before testing. After three submaximal warm-up repetitions and 1 min rest, concentric quadriceps and hamstring strength was tested at 60°/s through 0–90° knee flexion, followed by eccentric testing at 60°/s through 20–90° knee flexion, with five maximal repetitions for each mode^22^. Bilateral peak torque normalized to body weight (N/kg) was recorded. Strength testing was not repeated after the fatigue protocol to minimize injury risk.

Muscle fatigue was induced using the isokinetic dynamometer with 30 consecutive maximal knee flexion–extension contractions at 180°/s^23^. Isokinetic fatigue/endurance testing commonly uses angular velocities of 90–180°/s and approximately 30–45 consecutive concentric contractions in healthy and clinical populations^24^. An angular velocity of 180°/s has been widely used in dynamic fatigue protocols, including in PwMS^25,26^, while 30 repetitions provide a favorable balance between reliability and physiological interpretability of fatigue-related outcomes^25^. Participants received continuous verbal encouragement ("as fast and as hard as possible") and real-time visual feedback. Peak torque was recorded for each repetition, and the fatigue index was calculated as the percentage decline between the mean peak torque of the first and last three repetitions: [(initial-final)/initial]×100^27^.

### Statistical analysis

Analyses were performed using SPSS version 21. Normality and homogeneity of variances were assessed with the Shapiro-Wilk and Levene's tests, respectively. Normally distributed and categorical data were presented as mean±SD and percentages. Between-group comparisons used chi-square tests for categorical variables and independent t-tests for baseline values. A two-way repeated-measures ANOVA was conducted to examine the interaction (between-subjects factor: group [PwMS, HC]; within-subjects factor: time [pre-, post-fatigue]). Effect sizes for between-group comparisons were calculated using Cohen's d, with absolute values of 0.2, 0.5, and 0.8 interpreted as small, moderate, and large effects, respectively. Partial eta-squared (ηp^2^) was used for interaction effects, with values of 0.01, 0.06, and 0.14 interpreted as small, moderate, and large effects, respectively^16^. Significance was set at p<0.05.

## RESULTS

No significant between-group differences in demographic characteristics were observed (p>0.05), although the age difference approached conventional significance (p=0.057) (Table 1).

**Table 1:** Demographic characteristics of participants.

	PwMS (n=36)	HC (n=9)	p
Age (years)	41.33±8.83	47.67±7.95	0.057
Gender F/M (%)	30 (83.3)/6 (16.7)	8 (88.9)/1 (11.1)	0.681
BMI (kg/m^2^)	23.94±3.91	26.36±2.85	0.089
Foot dominance R/L (%)	36 (100)/0 (0)	9 (100)/0 (0)	1
Disease duration (years)	9.56±6.75	–	
EDSS	2.39±1.18	–	

Data are presented as number (%) of participants or mean±standard deviation, p<0.05, chi-square test or independent samples t-test, BMI: body mass index; EDSS: Expanded Disability Status Scale; L: left; R: right; PwMS: people with multiple sclerosis; HC: healthy control.

During gait, the swing phase was longer (p=0.032, d=0.82) and the double support phase shorter (p=0.017, d=-0.93) on the dominant side (DS) in PwMS than in HC. Baseline knee position sense was bilaterally similar between groups (p>0.05). Only concentric knee flexor strength was bilaterally lower in PwMS [p<0.05, DS: d=-0.91; Non-Dominant Side (NDS): d=-0.93]; during the fatigue protocol, total workload was bilaterally lower in both muscle groups in PwMS (p<0.05, extensors: DS, d=-1.01; NDS, d=-0.92; flexors: DS, d=-1.27; NDS, d=-1.28), with all statistically significant between-group differences representing large effects, though fatigue indexes were similar between groups (p>0.05) (Table 2).

**Table 2:** Baseline comparison of gait characteristics, proprioception, and muscle strength.

			PwMS (n:36)	HC (n:9)	Δ (95%CI)	p
Spatio-temporal parameters of gait						
	Step length (% str length)	DS	51.26±2.94	51.1±1.52	0.16 (-1.89, 2.22)	0.872
		NDS	49.01±3.33	48.9±1.52	0.11 (-2.20, 2.42)	0.922
	Stride length (m)	DS	1.25±0.23	1.16±0.28	0.08 (-0.10, 0.26)	0.365
		NDS	1.25±0.23	1.16±0.28	0.08 (-0.10, 0.26)	0.353
	% Stride length (% height)	DS	76.96±12.17	80.21±13.06	-3.25 (-12.53, 6.03)	0.483
		NDS	77.05±12.14	80.4±13.93	-3.35 (-12.74, 6.04)	0.476
	Speed (m/s)		1.2±0.24	1.16±0.31	0.03 (-0.16, 0.22)	0.737
	Cadence (steps/min)		116.14±13.71	120.81±8.66	-4.67 (-14.38, 5.04)	0.338
	Gait cycle duration (s)	DS	1.06±0.15	1.01±0.08	0.05 (-0.06, 0.15)	0.360
		NDS	1.06±0.15	1.01±0.08	0.05 (-0.05, 0.15)	0.338
	Stance phase (% cycle)	DS	60.26±3.37	61.81±4.88	-1.55 (-4.33, 1.23)	0.266
		NDS	61.5±2.4	62.12±2.18	-0.63 (-2.40, 1.15)	0.481
	Swing phase (% cycle)	DS	39.74±3.37	37.08±2.52	2.66 (0.24, 5.09)	**0.032** *
		NDS	38.5±2.4	37.88±2.18	0.63 (-1.15, 2.40)	0.481
	Single support phase (% cycle)	DS	38.16±3.24	37.92±2.21	0.23 (-2.08, 2.55)	0.840
		NDS	39.68±3.37	37.31±2.3	2.37 (-0.04, 4.77)	0.053
	Double support phase (% cycle)	DS	10.9±2.08	12.94±2.72	-2.04 (-3.70, -0.38)	**0.017** *
		NDS	10.9±2.29	12.01±1.99	-1.11 (-2.79, 0.57)	0.189
	Gait cycle symmetry index		91.79±7.23	95.29±6.67	-3.50 (-8.86, 1.86)	0.194
	Knee position sense (°)	DS	4.33±2.81	5.17±4.13	-0.84 (-3.17, 1.49)	0.471
		NDS	4.17±4.75	3.03±2.11	1.14 (-2.15, 4.43)	0.489
Knee muscle strength (N/kg) (60^⁰^/sec, 5 rep)						
	Concentric strength— extensor muscle	DS	109.89±40.84	138.22±43.38	-28.33 (-59.39, 2.72)	0.073
		NDS	109.94±42.88	128.44±36.1	-18.50 (-49.84, 12.84)	0.240
	Concentric strength— flexor muscle	DS	41.03±25.04	62.67±17.41	-21.64 (-39.53, -3.75)	**0.019** *
		NDS	40.11±23.45	60.44±14.28	-20.33 (-33.17, -7.50)	**0.004** *
	Eccentric strength— extensor muscle	DS	143.56±54.53	153.33±70.27	-9.78 (-53.21, 33.65)	0.652
		NDS	144.39±63.46	147.67±48.19	-3.28 (-49.05, 42.50)	0.886
	Eccentric strength— flexor muscle	DS	66.28±31.15	67±35.67	-0.72 (-24.80, 23.36)	0.952
		NDS	69.94±33.33	71.22±21.72	-1.28 (-24.95, 22.39)	0.914
Total work done (J) (180^⁰^/s, 30 rep)						
	Knee extensor	DS	1,743.28±655.87	2,374.56±487.51	-631.28 (-1,103.25, -159.31)	**0.01** *
		NDS	427.89±410.88	937.78±361.52	-588.89 (-1,070.29, -107.48)	**0.001** *
	Knee flexor	DS	1,676.11±668.46	2,265±500.3	-509.89 (-812.14, -207.63)	**0.018** *
		NDS	478.28±454	1,047.89±396.9	-569.61 (-903.26, -235.96)	**0.001** *
Fatigue index (%)						
	Knee extensor	DS	12±21.76	20.11±25.92	-8.11 (-25.09, 8.87)	0.341
		NDS	12.08±24.47	21.67±25.26	-9.58 (-28.09, 8.92)	0.302
	Knee flexor	DS	3.64±71.04	13.33±47.89	-9.69 (-60.31, 40.92)	0.701
		NDS	-31.28±114.14	20.11±47.13	-51.39 (-130.28, 27.50)	0.196

Data are presented as mean±standard deviation

* p<0.05, Independent samples t-test (between two groups). DS: dominant side; NDS: non-dominant side, J: joule; Δ: mean change (post-fatigue—pre-fatigue); 95%CI: 95% confidence interval; PwMS: people with multiple sclerosis; HC: healthy control. The bold values indicate statistically significant results.

Following the fatigue protocol, both groups showed increased cadence and reduced bilateral gait cycle duration (p<0.05); gait speed increased in PwMS (p=0.001), and the NDS double support phase decreased in HC (p=0.045). Knee position sense deteriorated bilaterally in PwMS (p<0.05) but only in the NDS in HC (p=0.035), with no significant group×time interaction overall (p>0.05) (Table 3).

**Table 3:** Gait characteristics and knee position sense following knee muscle fatigue protocol.

Outcome		Side	PwMS Δ (95%CI)	p^1^	HC	p^1^	T×G F/p^2^	ηp^2^
					Δ (95%CI)			
Spatio-temporal parameters of gait								
	Step length (%stride length)	DS	0.29 (-0.52, 1.09)	0.472	-0.09 (-1.43, 1.25)	0.882	0.198/0.658	0.005
		NDS	-1.12 (-2.54, 0.29)	0.116	0.09 (-1.25, 1.43)	0.882	0.714/0.403	0.016
	Stride length (m)	DS	0.02 (-0.01, 0.05)	0.219	0.00 (-0.05, 0.06)	0.896	0.239/0.627	0.006
		NDS	0.02 (-0.01, 0.05)	0.237	0.01 (-0.05, 0.06)	0.794	0.143/0.707	0.003
	% Stride length (% height)	DS	-1.38 (-7.20, 4.44)	0.633	-0.93 (-5.29, 3.42)	0.635	0.006/0.940	0.000
		NDS	1.21 (-0.61, 3.03)	0.187	-0.54 (-5.15, 4.06)	0.792	0.730/0.398	0.017
	Speed (m/s)		0.07 (0.03, 0.11)	**0.001** *	0.04 (-0.03, 0.12)	0.213	0.433/0.514	0.010
	Cadence (steps/min)		4.87 (1.66, 8.09)	**0.004** *	3.28 (0.26, 6.30)	**0.037** *	0.240/0.627	0.006
	Gait cycle duration (s)	DS	-0.05 (-0.09, -0.01)	**0.013** *	-0.03 (-0.06, -0.01)	**0.020** *	0.280/0.600	0.006
		NDS	-0.05 (-0.09, -0.01)	**0.010** *	-0.03 (-0.06, -0.00)	**0.031** *	0.308/0.582	0.007
	Stance phase (% cycle)	DS	0.18 (-0.69, 1.05)	0.679	0.13 (-2.96, 3.23)	0.923	0.002/0.967	0.000
		NDS	-0.48 (-1.31, 0.34)	0.242	-0.61 (-1.41, 0.19)	0.118	0.024/0.879	0.001
	Swing phase (% cycle)	DS	-0.23 (-1.08, 0.62)	0.589	0.98 (-0.21, 2.17)	0.094	1.874/0.178	0.042
		NDS	0.48 (-0.34, 1.31)	0.242	0.61 (-0.19, 1.41)	0.118	0.024/0.879	0.001
	Single support phase (% cycle)	DS	0.72 (-0.32, 1.77)	0.169	0.57 (-0.11, 1.24)	0.090	0.022/0.882	0.001
		NDS	-0.07 (-1.00, 0.86)	0.878	0.79 (-0.35, 1.93)	0.149	0.810/0.373	0.018
	Double support phase (% cycle)	DS	-0.23 (-0.73, 0.27)	0.353	-0.97 (-2.38, 0.44)	0.152	1.608/0.212	0.036
		NDS	-0.01 (-0.54, 0.52)	0.978	-0.49 (-0.96, -0.01)	**0.045** *	0.802/0.376	0.018
	Gait cycle symmetry index		1.01 (-0.70, 2.71)	0.240	2.19 (-1.72, 6.10)	0.233	0.395/0.533	0.009
Knee position sense (°)								
		DS	0.95 (0.07, 1.82)	**0.035** *	-0.04 (-4.32, 4.24)	0.983	0.628/0.432	0.014
		NDS	1.19 (0.33, 2.04)	**0.008** *	1.70 (0.15, 3.25)	**0.035** *	0.322/0.573	0.007

* p<0.05. Δ: mean change (post-fatigue—pre-fatigue); 95%CI: 95% confidence interval; p^1^: paired-samples t test within group; p^2^: time×group interaction by repeated-measures ANOVA; DS: Dominant Side; NDS: Non-Dominant Side; T×G: Time×Group; PwMS: people with multiple sclerosis; HC: healthy control. The bold values indicate statistically significant results.

## DISCUSSION

This study investigated baseline differences and the effects of knee muscle fatigue on gait characteristics and knee position sense in PwMS compared with HC.

At baseline, PwMS demonstrated a prolonged swing and reduced double support phase on the DS. Although previous studies generally report shorter swing and longer double support times as indicators of a stability-oriented gait strategy in MS^28^, those studies often included individuals with greater disability or examined bilateral parameters. Our findings are consistent with previous evidence from mildly disabled PwMS^5^ and suggest that subtle side-specific gait alterations may appear before the more global compensatory gait pattern typically observed at higher disability levels.

Despite these gait differences, baseline knee position sense did not differ between groups. This may be explained by the relatively young age and mild disability level of our sample, consistent with Iandolo et al., who reported clinically undetectable position sense deficits in early PwMS^6^. The numerically older age of the HC group should also be considered, as advancing age is associated with a longer double support phase. In contrast, PwMS showed significantly lower concentric knee flexor strength, while extensor strength was comparable between groups. This selective hamstring weakness is consistent with previous findings^29^ and may partly contribute to the altered swing-phase characteristics observed in our cohort.

### Knee muscle fatigue

During the fatigue protocol, PwMS generated lower total workload in both knee flexors and extensors, indicating reduced neuromuscular endurance; notably, this difference emerged despite the HC group being numerically older, and is therefore unlikely to be attributable to age. Fatigue indices, however, were statistically similar between groups and even numerically lower in PwMS—a counterintuitive result given the frequently reported motor fatigability in MS. This likely reflects the dependence of percentage-based indices on baseline torque capacity rather than intrinsic fatigue resistance. Consistent with this, Hameau et al. reported a lower concentric fatigue index in PwMS, yet when torque was normalized to maximal isometric peak torque, both groups finished at the same relative level (∼50% of initial capacity), attributing the difference to lower baseline torque rather than preserved fatigue resistance^30^. The same dissociation was reported by Lambert et al., who found that PwMS differed from controls in total work but not in the work-based fatigue index, leading the authors to conclude that total work was highly reliable in MS whereas the index was not^26^. Severijns et al. similarly cautioned that such indices may misrepresent fatigability when baseline capacity differs, noting that the greater absolute strength of controls may reduce muscle perfusion and increase peripheral fatigue, and that work-based fatigue indices derived from 30 maximal contractions at 180°/sec show only poor-to-moderate reliability in PwMS (ICC 0.36–0.51)^11^. Controls therefore had greater torque capacity and potential for absolute torque loss, whereas a smaller decline from a lower starting point could yield a comparable proportional fatigue index. Thus, a comparable or lower fatigue index should not be interpreted as preserved fatigue resistance, whereas the markedly reduced total work observed here provides a more direct indicator of impaired neuromuscular endurance.

Beyond this group-level discrepancy, the occasional negative fatigue index values warrant separate consideration. In some participants, initial torque was lower than final torque. Although continuous verbal encouragement and real-time visual feedback were provided, variability in achieving maximal torque during the initial repetitions cannot be excluded. PwMS have been shown to exhibit reduced torque steadiness, greater torque variability, and lower motor-unit discharge rates during voluntary knee extension contractions compared with HCs^31^. These alterations may contribute to less consistent torque production and partly explain lower initial torque values and the marked fatigue-index variability observed in our cohort.

Future studies may express concentric torque as a percentage of pre-fatigue maximal isometric torque^30^ and report total work as a complementary measure^26^, minimizing baseline-strength dependence and better representing fatigability in PwMS.

### Fatigue-induced changes in knee position sense and gait

Following the fatigue protocol, PwMS showed bilateral deterioration in knee position sense, whereas HC declined only on the NDS. The deterioration observed in HC is consistent with previous studies demonstrating impaired knee joint position sense following muscle fatigue in healthy individuals^32^. Potential mechanisms underlying fatigue-related proprioceptive deterioration include transient alterations in afferent feedback, muscle spindle sensitivity, and central sensory integration^32^. In PwMS, the bilateral pattern may also be influenced by the diffuse central nervous system (CNS) involvement of MS and altered interhemispheric communication^6^. Although the absolute post-fatigue deterioration in knee position sense was modest, changes of this magnitude may still be functionally relevant. Ozkan et al. reported a between-group difference of only 1.4° in knee repositioning error, yet this error was significantly associated with balance, gait, functional mobility, fear of falling, and fatigue in PwMS^4^. Therefore, the approximately 1° deterioration observed in the present study may have functional relevance despite its modest absolute magnitude.

For gait, both groups responded with increased cadence and decreased gait cycle duration, likely a generalized adaptive response prioritizing rhythmic stability over stride length. PwMS additionally increased gait speed. Although prolonged walking typically slows gait in PwMS^10^, our finding likely reflects the distinct fatigue induced: localized isokinetic knee fatigue elicits compensatory hip-flexor activation that offsets reduced knee-extensor output and maintains forward progression^33^. However, this proximal compensation occurred against bilateral proprioceptive decline, and any increase in walking speed under degraded sensory feedback may compromise dynamic stability and elevate fall risk rather than represent a safer adaptation.

The interpretation of these statistically significant gait changes should also consider measurement error. Published minimal detectable change (MDC) values for the G-Walk are 2.8 steps/min for cadence, 0.91 m/s for gait speed, 0.25 s for gait cycle duration, and 0.83–1.00% for double support^34^. Only the cadence increase exceeded the reported MDC in both groups, supporting a change beyond measurement error, whereas the other significant gait changes remained below these thresholds and should therefore be interpreted cautiously. However, these values were derived from healthy adults reassessed seven days apart and cannot be considered definitive thresholds for PwMS. Future studies should establish population-specific MDC values for G-Walk parameters in PwMS.

Despite these within-group changes, the absence of significant group×time interactions indicates that the pre- to post-fatigue changes in the assessed gait parameters and knee position sense did not differ significantly between mildly affected PwMS and HC. Nonetheless, more intense activity, heat sensitivity, or cumulative daily fatigue may reveal differences not captured by this protocol.

It is also important to distinguish performance fatigability from perceived fatigue. Similarities in some performance-based responses observed in the present study should not be interpreted as indicating comparable perceived fatigue in daily life, as these constructs may not necessarily parallel each other in MS^9^.

### Limitations

First, the PwMS in this study had mild disability and relapsing-remitting MS; therefore, we cannot generalize the results to PwMS with moderate to severe disabilities and other types of MS. Second, the relatively small number of HCs may limit the generalizability of the findings. Third, we evaluated only knee strength and position sense as lower-limb proprioception, did not reassess strength after fatigue, and did not include electromyography (EMG) or kinetic/kinematic gait analysis. Fourth, knee position sense was assessed at a single target angle (30°), which may not fully represent proprioceptive performance throughout the knee range of motion.

Finally, the large number of gait outcomes may have increased the risk of false-positive findings; therefore, significant gait results should be interpreted at the parameter level rather than as evidence of a generalized effect on gait.

## CONCLUSION

In mildly affected PwMS, baseline knee position sense was preserved while selective knee flexor weakness was evident. Similar fatigue indices despite lower total work should be interpreted cautiously because of the dependence of the Cybex fatigue index on baseline torque capacity. Following localized knee muscle fatigue, PwMS showed bilateral deterioration in knee position sense and increased gait speed, while both groups showed increased cadence and shorter gait cycle duration, with no significant group×time interactions. As proprioceptive acuity declined transiently after localized knee muscle fatigue, clinicians may consider performing balance- or coordination-demanding tasks before fatiguing knee exercise in PwMS.

## Data Availability

The datasets generated and/or analyzed during the current study are available from the corresponding author upon reasonable request.
